# Improving communication and promoting social inclusion for hearing-impaired users: Usability evaluation and design recommendations for assistive mobile applications

**DOI:** 10.1371/journal.pone.0305726

**Published:** 2024-07-17

**Authors:** Hyeonsu Kim, Heetae Hwang, Sojung Gwak, Jihyeon Yoon, Kyudong Park

**Affiliations:** 1 Department of Artificial Intelligence Applications, Kwangwoon University, Seoul, South Korea; 2 School of Information Convergence, Kwangwoon University, Seoul, South Korea; 3 SOVORO Inc., Seoul, South Korea; BRAC University, BANGLADESH

## Abstract

This study examines the usability of communication-assistive applications for hearing-impaired users, with a focus on enhancing user experience and promoting social inclusion. Although such applications have been developed and evaluated previously, interface designs that consider the intimacy needs of hearing-impaired users remain under-explored. We performed a comprehensive usability evaluation employing a mixed-method approach, which involved hearing-impaired individuals as well as field experts. The findings revealed areas for improvement in the design, validated the feasibility of implementing these applications, and emphasized the importance of incorporating the unique needs and preferences of hearing-impaired users. Furthermore, this paper discusses the importance of introducing guidelines and evaluation scales for the “Design for Emotion and Life Knowledge” levels to facilitate smooth and effective human–computer interactions. Such measures will promote the development of intelligent assistive technologies that reflect the qualitative needs of people with disabilities and contribute to social rights for hearing-impaired users. With the growing demands of artificial-intelligence-powered assistive technologies, the inclusion of individuals with disabilities in the design and research process is anticipated to increase. In future, studies should be conducted to blend the culturally shared experiences and emotional bonds expressed by users (having mild-to-severe hearing impairment) with the design and development process of assistive devices or services.

## Introduction

Recent studies suggest that advancements in science and technology have led to changes in society, prompting discussions on improving the quality of life through the use of intelligent services [1]. To date, several methods have been explored to enhance the quality of life of people with disabilities using intelligent assistive technologies [2,3], i.e., products or services that are designed to support hearing-impaired people and make them independent. Examples of currently available commercial assistive devices include cochlear implants and hearing aids for people with hearing impairment [4]. Despite advancements in assistive technologies, the perceived benefits of cochlear implants and hearing aids for people with hearing impairment may be negative because of difficulties in understanding speech in noisy environments [5]. Furthermore, the physical designs of hearing aids and cochlear implants inevitably expose users to undesirable situations.

Often, young people with hearing impairments are exposed to negative stigma from the visibility of hearing aids [6]. Other authors have reported that hearing-impaired students experience difficulties in social interactions with peers owing to vulnerabilities associated with language-based learning. Persistent gaps in academic achievement have been reported between students with and without disabilities [7–10]. Deaf students in Science, Technology, Engineering, and Mathematics (STEM) fields often face challenges due to a lack of professors experienced in working with Deaf individuals and an awareness of Deaf culture. This lack of access to necessary communication skills can result in Deaf students feeling dissatisfied or unable to study basic science and engineering subjects [11]. It is crucial to provide solutions to support Deaf students in these fields, including enhancing communication skills through general and specialized sign language instruction in university curricula [11]. Thus, further comprehensive studies on assistive technologies are required to improve the quality of life of students with hearing impairment by focusing on the relationships and needs of students throughout the educational process.

## Literature review

### Social misperceptions and challenges of assistive technologies

Recent studies have critically assessed the limitations and social challenges associated with conventional cochlear implants and hearing aids developed using intelligent assistive technologies [12,13]. A segment of this research indicates that the adoption of such technologies is often hindered by societal factors [14]. A phenomenon dubbed the "hearing aid effect" has been identified, wherein observers harbor negative attitudes towards individuals utilizing hearing aids [15]. This aversion often stems from the discomfort and stigma linked with visible body-interactive technologies in public spheres.

Profita [16] noted the substantial influence of sociocultural elements such as social stigma and prejudice, which sometimes compel users to forsake or conceal their assistive technologies. Rekkedal [17] further elaborated on this issue, mentioning the challenge posed by body-worn hearing devices, such as FM receivers, which tend to spotlight users, subsequently discouraging the use of these assistive communication devices. This spotlighting effect and the physical characteristics of these devices can notably dampen individuals’ enthusiasm to adopt assistive technologies. While it is true that the visibility of assistive technologies has traditionally been a source of stigma, recent trends show a growing acceptance of ear-worn technology, such as the widespread use of Apple AirPods by individuals without hearing impairments. Moreover, modern in-ear hearing aids are designed to be minimally visible. However, despite these positive trends, the stigma surrounding larger, body-worn assistive devices, such as cochlear implants, remains a significant barrier.

Intelligent assistive technologies emerge as potential alternatives to alleviate the social stigma encountered by individuals with hearing impairments. Generally characterized as an evolving range of intelligent applications devised to aid the disabled or elderly population, these technologies vary slightly in definition across different scholars [18]. Existing research [17] underscores a preference amongst hearing-impaired students for unobtrusive assistive devices. In light of this, real-time voice-caption interpretation mobile applications leveraging artificial intelligence (AI) integrated into smartphones offer a promising avenue for reducing the visibility stigma associated with traditional hearing aids.

### Usability testing of assistive communication mobile applications

An exploration into the user experience of assistive communication mobile apps reveals significant strides in this field. Elliot [19] undertook a usability evaluation of an automatic speech recognition application prototype, involving participants both with and without hearing impairments. This study aimed to enhance communication by transcribing spoken language into text messages, with findings suggesting a user-friendly interface and a positive role in fostering dialogues between individuals with varied hearing abilities.

Moreover, Samonte [20] introduced "BridgeApp", a pioneering communication tool offering features like text-to-speech, speech-to-text, and text-to-sign interpretation, aiming to facilitate communication for individuals with hearing impairments. User acceptance testing revealed a favourable reception of the application’s functionalities. Furthermore, Seita [21] spearheaded a collaborative design workshop and interviews focusing on user interaction with conversation partners through captioning applications, emphasizing the crucial role of optimal speech rate and eye contact in determining the preferences of individuals with hearing impairments.

Despite the promising developments showcased in these studies, a review of existing literature reveals a research gap pertaining to the specific needs of hearing-impaired students within educational environments. Currently, there exists a void in the establishment of design guidelines that cater to the unique requirements of this demographic in the context of assistive communication applications.

## Methodology

**Fig 1:**
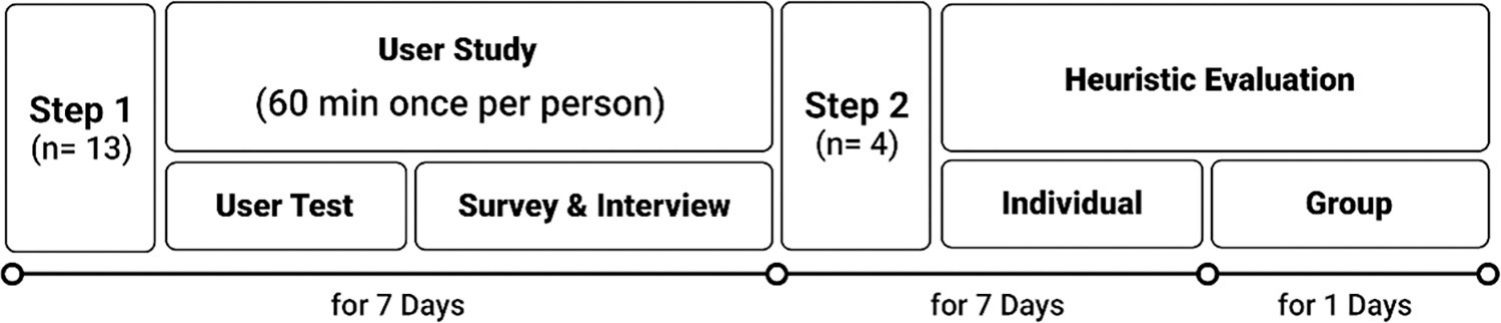
Research timeline of the two-stage study presented in this paper.

This study was designed to analyze the universal and pure usability issues in products and services for individuals with hearing impairment via comprehensive usability tests conducted using a two-step process (Fig 1). The first step, known as the user test, involved a representative user test, surveys, and interviews conducted with hearing-impaired participants. In the second stage, a heuristic evaluation was conducted by experts. According to a previous study [22], both universal and pure usability should be considered during the evaluation of website usability for people with hearing impairment. Universal usability refers to general usability issues that affect both disabled and non-disabled users, whereas pure usability indicates issues experienced by users with specific disabilities while using products or services. Involving HCI experts and developers in the process of developing products or services for individuals with hearing impairment is an efficient strategy to identify and assess universal usability issues.

In the first step, we focused on discovering usability issues through gathering opinions from hearing-impaired participants. In the second step, we identified the universal usability issues with the aid of HCI experts. This research methodology was adopted to overcome the limitations resulting from an insufficient number of participants with hearing impairment, while effectively identifying the universal usability issues.

**Fig 2:**
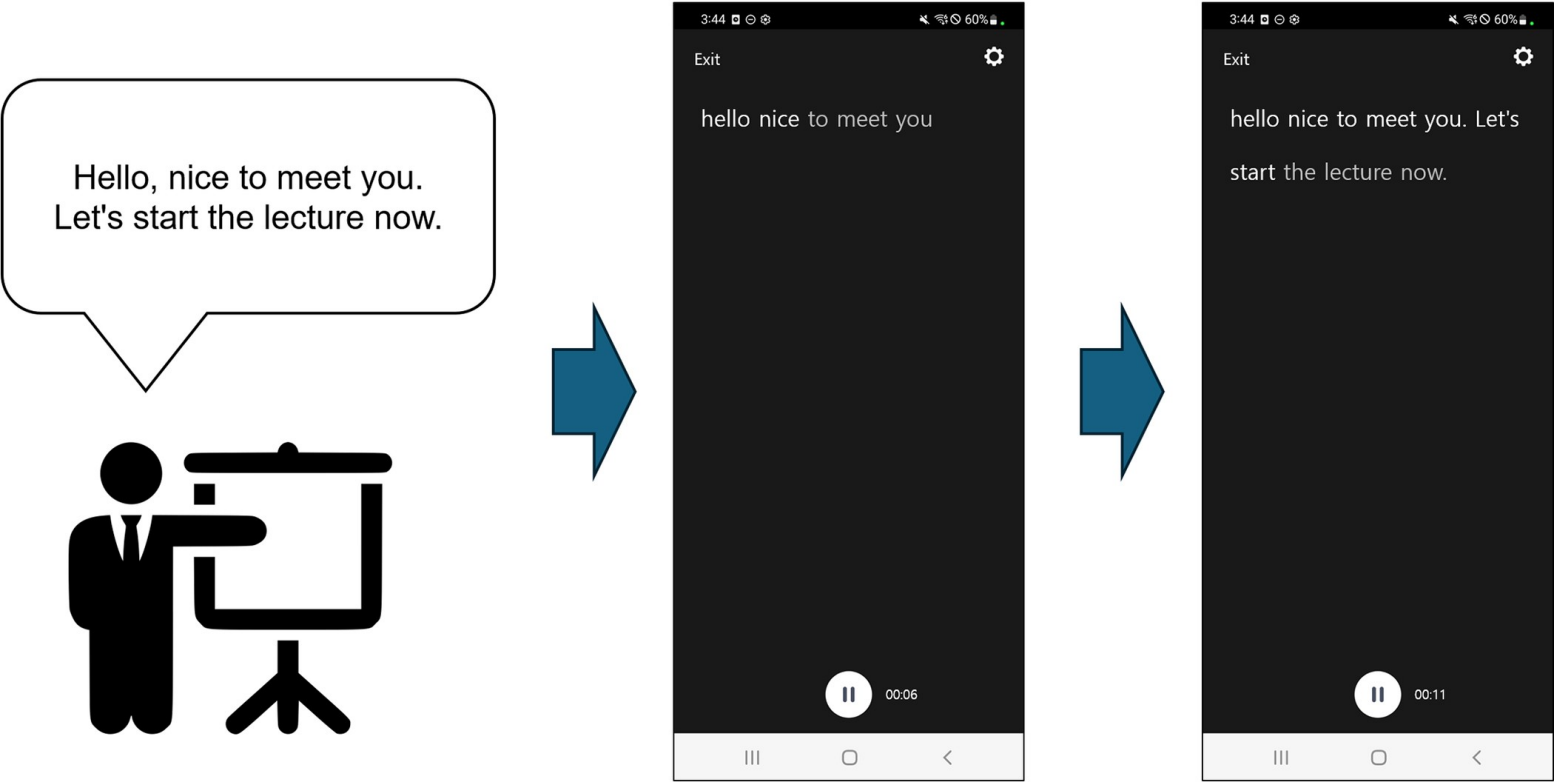
Target mobile application (Korean text in the app screenshots has been translated to English for reader’s understanding).

The mobile application developed by SOVORO Co., Ltd., which incorporates a speech-to-text function and is based on natural language processing, was used as the target application in our study. The specific version used was SOVORO (version 1.0.2), and it is currently available for free download on the Google Play Store (link: https://play.google.com/store/apps/details?id=kr.sovoro.app). This application recognizes the voice from a single speaker and converts it into gray text that can be displayed on a screen with an approximate 2-second delay, depending on network conditions. Once a sufficient amount of speech has been processed to understand the context, the text is either modified or confirmed. At this stage, the text color changes to white (Fig 2). This target application also supports various features such, as voice caption generation, voice caption storage, and viewing stored captions.

The interface of this mobile application comprises screens regarding display, stored-interpretation confirmation, and account management. On the caption-interpreting screen, users can generate real-time interpretations based on surrounding voices or stop caption generation by touching a button on the app screen at the desired time. The interpretation confirmation screen allows users to view stored voice captions in reverse chronological order, and the application automatically records voice-generated captions, to provide a playback feature.

## User study

### Participants

Since hearing-impaired people in the age group of 19 to 34 years are the primary target users of AI-based communication apps, we selected thirteen hearing-impaired people in this age group to participate in user evaluation in this study. All participants were Korean individuals who primarily spoke Korean. The participants could communicate using hearing aids, sign language, or writing aids. These participants were recruited from various educational institutions in Korea, where they were enrolled as students to obtain a degree or qualification to secure employment or develop skills. Individuals facing difficulty in reading and understanding captions were excluded from the study. The average age of the participants was 24.7 (standard deviation, SD = 4.81); seven participants regularly used cochlear implants, three used hearing aids for communication, and the remaining three communicated through sign language, writing, or lip reading and did not use hearing aids.

### Ethical considerations

This study procedures approved by the Institutional Review Board of Kwangwoon University (approval number: 7001546-20220804-HR(SB)-007-05), and the hearing-impaired participants were informed as well. The researcher provided participants with an ethical consideration guide and a prepared research explanation document. The researcher informed the participants of the expected side effects and benefits of the experiment, and the participants were informed that they could voluntarily discontinue their participation or receive a break whenever they wanted. All the hearing-impaired participants provided written informed consent prior to participating in the study. The participants were provided with pre-prepared laptops to communicate by typing or freely asking questions with the assistance of a professional interpreter. After completing the party evaluation procedure, the participants were paid a participation reward of KRW 70,000.

### Procedure

In October 2022, the user test was conducted under a simulated classroom environment in an office located in Seoul, South Korea. Before the main procedure, a pilot test was conducted with a young, nondisabled individual to prevent errors during the user evaluation. For each participant, the entire process, including task analysis, surveys, and interviews, was completed within approximately 60 min. The evaluation app was preinstalled on a Galaxy S21+ (6.7-inch) smartphone provided to the participants. The script material prepared for the mock lecture was designed for individuals with higher education, and the lecture was conducted by an experimenter, who read the script. The script was based on a textbook for a general elective subject taught at a university in Seoul, Korea. The topic of the script is the importance of understanding customer needs and an explanation of explicit and potential needs.

### Experimental tasks

For the user tests, the participants were instructed to perform typical tasks using the communication app and to report any difficulty encountered during the process. Prior to conducting the test, a literature review related to user tests of communication apps was conducted to derive the tasks for the experiment. Subsequently, two researchers majoring in HCI finalized an agreement based on the usability principles presented by Nielsen (1994) [23] and selected the representative tasks to be presented to the participants and the corresponding task instruction elements, as shown in Table 1.

**Table 1:** List of tasks presented to the study participants.

General tasks	Specific tasks
Start Interpreting	1. Find the Start Interpretation button on the home screen 2. Press the Start Interpretation button on the home screen
Experience Interpreting	3. Experience the process of interpreting the simulated lecture for 3 minutes
Ending and Saving Interpretation	4. Press the Interpretation End button on the Interpretation Progress screen 5. Write and save the interpretation title
Confirming Interpretation	6. Find your saved interpretation files on the home screen

The user test involved providing participants with a smartphone with a pre-installed communication app and assigning tasks to be completed using the app. The tasks were structured as follows: starting an interpretation, experiencing the interpretation, ending and saving the interpretation, and confirming it. During the interpretation experience task, participants observed a mock lecture delivered by the researcher and experienced the app’s functions while watching the voice-to-caption interpretation displayed on the mobile screen.

### Survey and interview

During the survey and interview stages, the experimenter collected the participants’ responses to prepare usability evaluation questions and conducted semi-structured interviews to gather their opinions after completing the task analysis. Upon completing the task analysis, the participants were given a prepared survey to which they responded. The researcher created an operational definition of the measured variables and structured the survey items based on previous studies (Table 2).

**Table 2:** Measurement variables used in this study and their operational definitions.

Measured variables	Operational definition
Trust	An attitude of accepting and relying on the outcome of interpretation.
Perceived Usefulness	The degree to which one subjectively believes that one’s efficiency can be improved through the result of interpretation.
Continuance Intention	The intention to continue to use assistive mobile applications in the future.
Satisfaction	Favorable response according to the pleasant emotion obtained from interaction with assistive mobile applications.
Overall Usability	The series of experiences to efficiently and effectively perform desired tasks according to one’s intentions and purposes.

The measured variables were trust, perceived usefulness, continuance intention, and satisfaction. The overall usability was measured using a system usability scale [24]. The following section provides a detailed explanation of the measured variables. After responding to the survey, participants were interviewed about the efficiency, effectiveness, barriers, and suggestions for improvements related to the communication app interface for individuals with hearing impairment.

### Dependent measures

#### Trust

Shankar et al. [25] has defined trust as "the belief by one party about another party that the other party will behave in a predictable manner" and argued that customer trust affects satisfaction, repurchases, and loyalty. In automated systems, trust influences the extent to which people accept and rely on a system [26]. Jian et al. [27] asserted that understanding the relationship between trust in computerized systems and their usage requires an effective measurement of trust. In this study, trust was defined as the attitude of accepting and relying on the outcomes of real-time speech interpreting systems. Appropriate items from the reported trust survey of [27] were selected to assess the usability of communication apps. Four items were selected and presented to the participants on a 7-point Likert scale (1 = strongly disagree, 7 = strongly agree).

#### Perceived usefulness

Perceived usefulness refers to the subjective belief that a system or service can enhance efficiency. Ajzen [28] argued that the perceived usefulness of a product or service is linked to continued usage, whereas Bhattacherjee (2001) [29] demonstrated that perceived usefulness is a critical factor in users’ continued use of a service. In this study, perceived usefulness was defined as the extent of subjective belief in the ability of real-time speech interpreting systems to enhance efficiency. The survey items reported by Bhattacherjee (2001) [29] were adapted, modified, and presented to the participants on a 7-point Likert scale.

#### Continuance intention

Continuance intention is measured to infer users’ continuous behavior in various fields [30]. Bhattacherjee (2001) [29] defined continuance intention as "the intention to continue using an information system" (p. 359). In related work [31], continuance intention is "a mental state reflecting an individual’s decision to repeat her current behavior" (p. 138). In a study related to e-learning systems, Chang (2013) [30] defined continuance intention as "the intention to use the system in the future" (p. 42). Here, continuance-intention is defined as the intention to continue using assistive mobile applications in the future. We adopted the survey items used by Bhattacherjee (2001) [29] to measure continuance intentions.

#### Satisfaction

Westbrook (1981) [32] defined satisfaction as consumers’ emotional responses to the evaluation of their experience obtained from the use, consumption, and ownership of a specific product or service. Oliver (2014) [33] referred to satisfaction as a reaction to the fulfillment state provided by a product/service feature or the product/service itself in terms of consumption. Wixom (2005) [34] conceptualized satisfaction as “a degree of favorableness with respect to the system and the mechanics of interaction.” Here, based on previous research, satisfaction is defined as the favorable response of users based on pleasant emotions obtained from interacting with assistive mobile applications. We adopted the survey items proposed by Wixom (2005) [34] and used a 7-point Likert scale.

#### Usabilit

Usability refers to how efficient a service is in achieving what it wants, whether there is no significant difficulty in using it, and whether mistakes can be easily and quickly resolved. Brooke (1996) [24] mentioned usability as a universal appropriateness for a specific purpose and proposed a system usability scale (SUS) as a measure to evaluate usability. Over the past few decades, the SUS has been one of the most widely used methods among various shorthand methods for evaluating usability, comprising 10 questions each. The survey questions were designed to be answered on a 5-point Likert scale (1 = strongly disagree, 5 = strongly agree), and scores were calculated using a specific method. According to Sauro (2016) [35], who comprehensively reviewed studies using SUS, scores corresponding to the top 10, 30, and 50% were approximately 80.8, 74.1, and 68 points, respectively, allowing the inference of the overall usability level of products. Here, overall usability is defined as a series of experiences in which users efficiently performed desired tasks according to their intentions and purposes using assistive mobile communication applications. The SUS was used to measure its scores and levels.

### Data collection and processing

In data collection and processing based on party evaluation, statistical methodologies were used only for reference purposes because of the lack of participants. In this study, descriptive statistics, such as means and variances within survey questions or participant groups, were utilized. The differences between survey questions and between the hard-of-hearing and severely-hearing-impaired groups were examined by comparing the error bars of the statistics at the 95% confidence interval. The interview results were transcribed, and two researchers agreed to classify them by topic, following the qualitative data analysis method proposed by Braun (2006) [36].

### Results

#### Survey

The survey results revealed that the average response score for satisfaction among the 13 participants with hearing impairment was 4.38 (SD = 1.40) out of a maximum of 7 points (Table 3). Trust received an average score of 4.38 (SD = 1.45). Continuance intention scored an average of 4.37 (SD = 1.69) and the average perceived usefulness score was 4.35 (SD = 1.41).

**Table 3:** Survey results obtained using a 7-point Likert scale.

(N = 13)		
Measurement variables	Mean	SD
Satisfaction	4.38	1.40
Trust	4.38	1.45
Continuance Intention	4.37	1.69
Perceived Usefulness	4.35	1.41

According to the system usability scale calculation, the average overall usability score was 66.73 on average, with a SD of 17.42. Although the response scales presented to the participants were different, the SD (= 17.42) of the overall usability score was higher than that of the other variables. According to Sauro (2016) [35] and Bangor (2009) [37], the usability level of the communication app evaluated using SUS was slightly below the top 50%, that is, between acceptable (okay) and good.

#### Interviews

Semi-structured interviews were conducted to explore the efficiency, effectiveness, barriers, and suggestions for improving the communication app interface designed for people with hearing impairment. All the participants (N = 13) responded to the interviews, and their responses were typed, recorded, and qualitatively analyzed by the experimenter. The analytical process included identifying explicit or latent meanings and categorizing common themes from recurring patterns in the participants’ responses. The responses were classified into four categories: ’consideration of diverse usage contexts; accuracy requirements for trust, usefulness, and expectations; and the need for an integrated system.

#### Consideration of diverse usage contexts

The consideration of diverse usage contexts implies the need for effective voice recognition in situations beyond typical environments such as noisy settings, multiple people speaking simultaneously, and the use of dialects or technical terms. Representative opinions from the interviews included “the need for technology to collect various languages, dialects, and technical terms and incorporate them into the AI (P3),” “the likelihood of using the app in group assignments or café meetings outside of lecture environments (P6),” “curiosity about the app’s translation capabilities in noisy environments (P11),” and “the desire to know whether the app can recognize speech when a friend with hearing impairment has poor pronunciation (P11).” The suggestions derived from this theme were the need for voice recognition apps to learn corpora of foreign languages, dialects, and technical terms, and to maintain high accuracy in noisy environments.

#### Necessity for accuracy to ensure trust

Regarding the necessity of accuracy to ensure trust, it was commonly identified through interviews that the participants demanded improvements in the real-time translation accuracy of communication apps. For individuals with hearing impairment, it is particularly challenging to compare and capture sound information in real time to verify whether speech is being accurately interpreted in captions. This highlights the importance of trust in a system. Representative interview content included statements such as "I hope that translations of speech during real-time translation processes are accurate. Hearing-impaired individuals cannot determine the accuracy themselves, and thus, trust issues regarding accuracy repeatedly arise (P3)," "I wish the accuracy was higher when recognizing (words) in real-time (P4)," and "Speech interpreting needs better accuracy. While individuals with mild hearing loss may find (errors) acceptable, it is difficult for hearing-impaired individuals to correct (errors) as they cannot hear at all (P2)." Through this theme, it was possible to identify the needs of individuals with hearing impairment for whom translation errors in communication applications can be more critical and directly impact trust, thus emphasizing the need for improved speech translation accuracy.

#### Perceived usefulness and expectations

Regarding perceived usefulness and expectations, participants reported that the communication app used in this study was functionally superior to previously used apps and tended to highly appreciate the app’s ability to quickly translate speech into text. Participants with hearing impairment expressed the need for useful communication support systems in the learning environment. Some participants (P11 and P2) expressed their expectations of improved learning environments through the application used in this study. They shared comments such as, "It would be great if the app and hearing devices could be linked during remote classes, as I often attend lectures using both (P11)," "When I first used it, I liked that the sentences were generated more quickly in this app than in previous communication apps. The speech captions were easy to understand and read quickly, which was great (P12)," and "It seems like it would be very helpful during online lectures (P2)." These comments confirm that some participants held positive attitudes toward the usefulness of the speech recognition app used in the experiment.

#### Need for an integrated system

In the context of the need for an integrated system, participants consistently expressed their desire for additional convenient features when utilizing speech recognition apps in educational environments. This indirectly revealed the participants’ expectations that speech recognition apps would improve their learning environment. They also identified the potential need for a comprehensive learning support system tailored for individuals with hearing impairment rather than a simple communication assistance system. Representative responses included, "It would be great if we could add supplementary meanings to specific words in a separate notepad, and be able to open and view them by tapping on them (P7)."It would be nice if the translation content could be shared for use in learning’(P9). "It would be helpful to study if there was a feature to search for unknown content immediately during learning (P10).". Based on these findings, there is a need to construct an integrated system that supports and manages learning tailored to student characteristics, provide a high-quality user experience, and improve the learning environment for individuals with hearing impairment.

## Heuristic evaluation

### Participants

In the heuristic evaluation procedure, four experts in software or information technology, were selected for identifying and analyzing the usability issues of the target app. Among these four experts, three had relevant research experience in user experience and HCI fields, and one had expertise in mobile application development.

### Method

In the second stage of the study, a heuristic evaluation was conducted. Heuristic evaluation is a method of evaluating usability issues based on prior research and expert knowledge. It rapidly identifies usability issues using standardized processes and principles based on professional and technical insights. The heuristic evaluation primarily employs the 10 usability principles proposed by Nielsen (1994) [23] to discover instances where the target’s usability is violated. These principles can be used to evaluate usability with intuitive principles, irrespective of the type of target [38]. Moreover, a heuristic evaluation can be conducted at any stage after prototype design during the development process and is thus an efficient strategy [39]. In this study, the heuristic evaluation was conducted by going through a process of individual evaluation and group evaluation. An individual evaluation means finding usability issues by each expert, and a group evaluation means merging usability issues as a group discussion and agreeing on the severity of their issues among experts [40]. In this study, participants were provided mobile devices (Galaxy S21+) with the target app installed. During the evaluation process, the participants could freely explore the target app using the device. The entire process of the heuristic evaluation was completed in November 2022.

### Individual evaluation

During the individual evaluation, each expert performed tasks to identify usability issues based on the 10 usability principles proposed by Nielsen (1994) [23]. In this process, the experts conducted a weeklong assessment to discover instances of violated usability principles such as providing appropriate feedback to users regarding progress (system status visibility), distracting users with unnecessary elements (aesthetic and minimalist design) and preventing error-prone conditions (error prevention). The experts reported their individual findings in a shared online workspace.

### Group evaluation

During the group evaluation, usability issues discovered through the individual evaluation were categorized, and a consensus was reached on their severity. The group evaluation was conducted for four hours in November 2022 at a research laboratory located at a University in Seoul. The experts assessed severity using a five-point Likert scale (ranging from 0 to 4 points). Under the severity score criteria, the lowest score of 0 indicates no error, whereas a score of 1 indicates a minor error that does not cause inconvenience to users. A score of 2 represents an error causing discomfort for users, and 3 points denote a serious error causing significant inconvenience for users. The highest score of four points signifies a critical error that urgently requires resolution owing to its detrimental impact on usability. During the process of assigning severity to each usability violation, four researchers performed individual assessments and shared their severity ratings for each case. Subsequently, a group discussion was held, and the procedure was repeated until a unanimous agreement was reached on the severity scores.

### Results

The heuristic evaluation (which merged individual and group evaluation) indicated 13 instances of usability principles violations. The highest number of violations (five) occurred for the principles of flexibility and efficiency of use. For the other usability principles, only one or two usability issues were identified. Specifically, two usability violation instances were found in the principles of error prevention, and one was discovered in the principles of aesthetic and minimalist designs. Moreover, only one usability violation instance was identified in each of the usability principles of user control and freedom, visibility of system status, “help users recognize, diagnose, and recover from errors,” and recognition rather than recall. No usability violation cases were found in the principles of matching between the system and the real world, consistency and standards, or help and documentation (Table 4).

**Table 4:** Results of heuristic evaluation.

Usability Principle	Instance	Severity Score
Flexibility and Efficiency of Use	1) “Can’t go back to record”	3
	2) “The process of deleting interpretation files is complicated”	3
	3) “It takes a long time to cancel the interpretation function in the application”	2
	4) “Unable to select or edit saved voice-to-caption interpreting text by tapping”	3
Error Prevention	5) “There is no warning window to double-check if you accidentally hit the delete button.”	4
	6) “Interpretation files can be continuously created with the same name”	2
Aesthetic and Minimalist Design	7) “The application’s home screen is supposed to display up to 5 translation files, but the rest of the screen is just blank”	1
Visibility of System Status	8) “After saving the file, there is no clear signal that voice-to-caption interpreting complete on the file”	3
Help Users Recognize, Diagnose, and Recover from Errors	9) “In the case of foreign language interpretation, it is possible to create real-time captions during interpretation, but the interpretation result is lost in the final conversion script after saving.”	4
Recognition rather than Recall	10) “It’s hard to find the interpreter start button intuitively”	2

The severity score assessment in the group discussion showed one severe usability violation instance in the principle of “help users recognize, diagnose, and recover from errors” (4 points). In this case, the interpretation results in the final converted script disappeared after the English interpretation was saved, and no measures were available for the users to diagnose or recover from the errors. The same result was confirmed in the principle of error prevention, where a high severity was assigned to the violation caused by the lack of a confirmation message when the user accidentally pressed the delete button. For other usability principles, the severity scores ranged from 0 to 3 points.

## Discussion

### User study

An integrated analysis of the user study results indicated that the participants responded favorably to the user experience of the communication app in supporting their learning environment. In addition, the qualitative results suggested that assistive mobile applications for hearing-impaired individuals require various functions to support learning and communication. This result indicates such applications should be based on a system that comprehensively manages and supports the social needs of people with disabilities.

Conversely, the negative responses mainly originated from users with severe hearing loss. This study’s participants ranged from people with mild hearing impairment to those with severe hearing loss. Because severe hearing loss requires more effort to listen to sounds than mild hearing loss, in the user test, some of the participants with severe hearing loss may have found it challenging to verify whether their real-time interpretation displayed on the mobile screen matched actual voice. During the user test as well, the participants with severe hearing loss were placed in a situation where they had to rely on voice-to-caption to judge the interpretation’s accuracy without taking actual voice. These observations indicate that assistive mobile applications should be tested by considering the issues faced by people with complete (or almost complete) hearing loss, as they may be more sensitive to errors. On the real-time interpretation manner, trust of the hearing impaired may also be correlated with the severity of the hearing loss. Such factors may have led to a bias in the responses to surveys. These results should be considered in future studies.

### Heuristic evaluation

When considering the numerous usability violation instances (five instances) found in the principles of flexibility and efficiency of use, most cases were rated with a medium level of severity (1–3 points) rather than a critical or catastrophic severity. This rating suggests that the issues were not related to functional defects in the app. Rather, most of the issues resulted from the lack of convenient features, such as file selection, which are commonly provided in other apps. These shortcomings need to be mitigated in the near future to develop more efficient and user-friendly assistive apps for hearing-impaired people. Although only one usability violation instance was identified for the principles of “help users recognize, diagnose, and recover from errors” and error prevention, their severity appeared critical. Notably, even a single usability violation instance of high severity can lead to detrimental outcomes for users. Therefore, the evaluated communication assistance app should be further improved to prioritize addressing the identified high-severity usability issues. The overall expert consensus was inclined toward incorporating certain improvements in the target app to widen its usability among users suffering from varied degrees of hearing loss. Notably, no usability violation cases were detected in terms of consistency and standards between the system and the real world or in the provision of help and documentation. This result was obtained possibly because of the low difficulty or lack of elements that users may find challenging in terms of the information architecture. However, the main user group (hearing-impaired) and experts may have different human factors and disability experiences, which may have resulted in additional usability issues that the experts could not identify. Therefore, a complementary interpretation of the user evaluation results is necessary.

### Comprehensive discussion

#### Direction of assistive technology development

The hearing-impaired participants emphasized their desire for communication apps to manage and support their difficulties in learning environments and daily life rather than merely receiving assistance for their disability. This perspective could be interpreted as a simple demand for learning support features as well as related to the requirement of customizable assistive technology devices and services. Based on the results obtained in previous studies, the findings obtained in our study suggest that assistive technology should be considered a customized tool and methodology that manages and coordinates social participation based on individual needs; and its usability extends beyond being a simple functional support for teaching, learning, and assisting in daily activities. Therefore, we propose the need for an integrated system that serves as a social mediator and enables people with disabilities to perform daily life activities as well as participate in social activities in their desired manner. The overall usability evaluation results showed a score that far exceeds the borderline of 50.9 points (“OK.”) [37]. The target group of our study were people with severe disabilities, and we evaluated the usability of communication-assisting applications, the evaluation of some usability issues may appear more critically biased when compared to the issues faced by general users. These results highlight the need for new usability evaluation measures and criteria for users with disabilities.

### Requirement of deaf extra linguistic knowledge

The hearing-impaired participants displayed unexpected behaviors during the behavioral observation and interview process. Several participants expressed the desire to ensure that the developers and researchers of the evaluated app fully understood the culture and experiences of the hearing-impaired population (P4: "If non-disabled people want to better understand the position of hearing-impaired people, then they should wear earplugs for a week, from waking up to going to sleep, without taking them off, and try various experiences, including attending lectures") or expressed regrets related to this matter (P3: "I think the features and user experience would have been different if hearing-impaired people or people with disabilities had also been involved in creating this app"). Additionally, during the behavioral observation, participants expressed concerns related to the possibility of replacing stenographers, who usually accompany them, with AI technology if the communication app is commercialized. According to previous studies, this concern can be interpreted as the need to reflect the shared experience of deaf extra linguistic knowledge (DELK) in the development process and user-experience evaluation of future mobile-based assistive communication applications. Woolfe (2019) [41] highlighted the importance of empathetic and culturally competent interpreters, particularly sign language and writing interpreters, for hearing-impaired people. These reported studies indicate that the interpretation experience is mediated by the process of sharing the hearing-impaired individual’s discriminatory experience and culture beyond linguistic knowledge and support and that DELK should influence the interpretation process.

The evaluation results suggest that while using the real-time speech interpreting app, the hearing-impaired participants possibly focused more on the adverse effect of such assistive apps on the mechanics of their relationship with stenographers rather than on the performance or interface elements of the app and its ability to improve their quality of life. This implies that the participants largely compared the app with stenographers (who are a usual source of social interaction and social support for hearing-impaired people), which may have influenced the survey responses and interviews. However, to date, the intimacy between hearing-impaired users and stenographers has been rarely considered during the development and evaluation of communication-assisting apps. In the future, it will be necessary to develop assistive technology devices and services that provide communication functions as well as social support for hearing-impaired users. Additionally, the evaluation results obtained in the present study indicate the importance and feasibility of introducing scales or guidelines for evaluating the DELK levels from the perspective of HCIs. These results collectively demonstrate the social value of intelligence assistive technologies that reflect the qualitative needs of the disabled.

## Conclusions

In summary, we explored the usability and effectiveness of communication-assisting mobile applications for hearing-impaired users. We conducted a comprehensive usability evaluation and interpreted the feedback provided by hearing-impaired participants as well as field experts to identify areas that can be improved for a better user experience. The increasing interest in utilizing intelligent assistive technologies, particularly those employing AI, highlights the need for more inclusive research methodologies that involve disabled individuals for designing and developing products and services. The results obtained in this study emphasize the importance of considering the unique needs and experiences of hearing-impaired users, particularly when developing interfaces that foster intimacy and social support.

It is, however, pertinent to acknowledge the geographical limitations of our participant pool, confined to select regions in East Asia, which potentially restricts the broader applicability of our findings due to varied socio-cultural dynamics among the global hearing-impaired populace. To substantiate these preliminary findings, it is recommended that future studies cast a wider net, encompassing a greater diversity in sample size and cultural backgrounds, and socio-economic statuses, particularly including groups from developing countries. This inclusion is crucial as the results may significantly differ from the population samples typically used.

Ultimately, the insights and recommendations emanating from this research hold the promise to lay a solid groundwork for the evolution of innovative, inclusive assistive tools and services. By centering the needs and experiences of hearing-impaired individuals, a collaborative synergy between researchers and developers can be fostered, thereby elevating the societal impact and transformative potential of intelligent assistive technologies, while concurrently enhancing the life quality of individuals grappling with disabilities. As we look towards the future, it is critical that subsequent research endeavors delve into the communal experiences and emotional connections fostered when individuals spanning a spectrum of hearing loss engage with assistive devices and services as mediums of social interaction. Analyzing these elements through the lens of Human-Computer Interaction (HCI) will pave the way for the development of assistive technologies that are not only more effective but also attuned to the genuine needs of their intended users.

## Supporting information

S1 File Survey data. (XLSX)
